# Supplementary material for: The causal relationship between the human gut microbiota and pyogenic arthritis: a Mendelian randomization study
Source: Front Cell Infect Microbiol. 2024 Nov 26;14:1452480. doi: 10.3389/fcimb.2024.1452480 (PMC11629706; doi:10.3389/fcimb.2024.1452480)
Supplement: Supplementary file 1 [file DataSheet1.zip › mendelian test/ebi-a-GCST90027707.csv_leave_one_out.pdf]

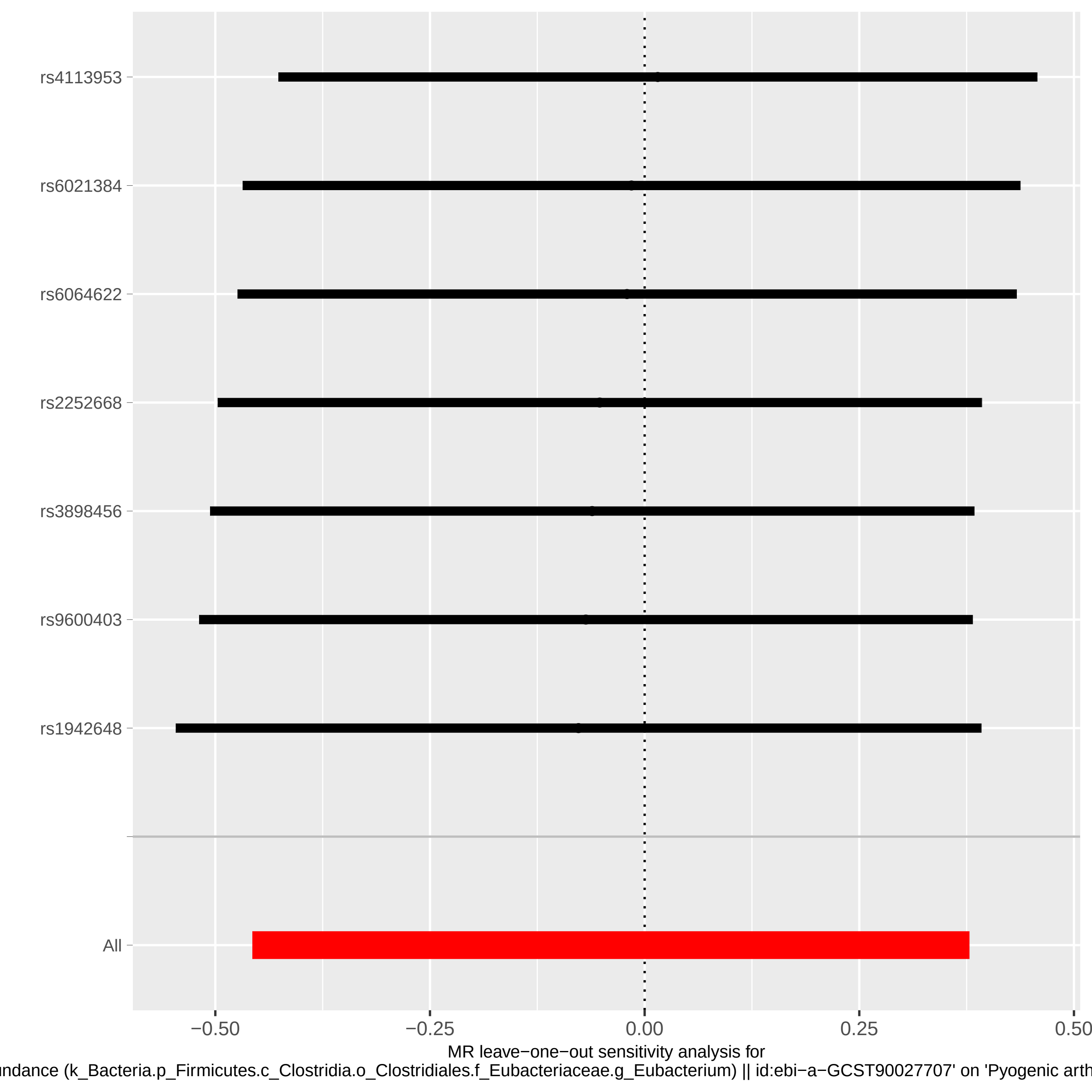

rs4113953
rs6021384
rs6064622
rs2252668
rs3898456
rs9600403
rs1942648
All
−0.50
−0.25
0.00
0.25
0.50
MR leave−one−out sensitivity analysis for
undance (k_Bacteria.p_Firmicutes.c_Clostridia.o_Clostridiales.f_Eubacteriaceae.g_Eubacterium) || id:ebi−a−GCST90027707' on 'Pyogenic arth
